# Detection of patient setup errors with a portal image – DRR registration software application

**DOI:** 10.1120/jacmp.v12i3.3492

**Published:** 2011-02-18

**Authors:** Kenneth Sutherland, Masayori Ishikawa, Gerard Bengua, Yoichi M. Ito, Yoshiko Miyamoto, Hiroki Shirato

**Affiliations:** ^1^ Hokkaido University Graduate School of Medicine Sapporo 060‐8648 Japan; ^2^ Hokkaido University Hospital Sapporo 060‐8648 Japan

**Keywords:** patient setup, image registration, portal imaging, mutual information

## Abstract

The purpose of this study was to evaluate a custom portal image — digitally reconstructed radiograph (DRR) registration software application. The software works by transforming the portal image into the coordinate space of the DRR image using three control points placed on each image by the user, and displaying the fused image. In order to test statistically that the software actually improves setup error estimation, an intra‐ and interobserver phantom study was performed. Portal images of anthropomorphic thoracic and pelvis phantoms with virtually placed irradiation fields at known setup errors were prepared. A group of five doctors was first asked to estimate the setup errors by examining the portal and DRR image side‐by‐side, not using the software. A second group of four technicians then estimated the same set of images using the registration software. These two groups of human subjects were then compared with an auto‐registration feature of the software, which is based on the mutual information between the portal and DRR images. For the thoracic case, the average distance between the actual setup error and the estimated error was 4.3±3.0 mm for doctors using the side‐by‐side method, 2.1±2.4 mm for technicians using the registration method, and 0.8±0.4 mm for the automatic algorithm. For the pelvis case, the average distance between the actual setup error and estimated error was 2.0±0.5 mm for the doctors using the side‐by‐side method, 2.5±0.4 mm for technicians using the registration method, and 2.0±1.0 mm for the automatic algorithm. The ability of humans to estimate offset values improved statistically using our software for the chest phantom that we tested. Setup error estimation was further improved using our automatic error estimation algorithm. Estimations were not statistically different for the pelvis case. Consistency improved using the software for both the chest and pelvis phantoms. We also tested the automatic algorithm with a database of over 5,000 clinical cases from our hospital. The algorithm performed well for head and breast but performed poorly for pelvis cases, probably due to lack of contrast in the megavoltage portal image. The software incorporates an original algorithm to fuse portal and DRR images, which we describe in detail. The offset optimization algorithm used in the automatic mode of operation is also unique, and may be useful if the contrast of the portal images can be improved.

PACS numbers: 87.55.Qr, 87.57.nj

## I. INTRODUCTION

Traditionally doctors have judged patient setup errors by viewing portal images alongside planning digital reconstructed radiograph (DRR) images, either with paper printouts, films on a light board, or on a computer terminal. Estimation of the error is made by measuring the distance from the isocenter to anatomical structures (usually bones) visible in both images.^(1)^ However, the process is often inaccurate, with errors between 5 and 10 mm being reported.^(2)^

Fully and semi‐automatic methods based on electronic portal imaging devices (EPIDs),^(3)^ implanted fiducial surrogate markers imaged with kilovoltage fluoroscopy,^(4)^ on‐board imager (OBI, Varian Medical Systems, Palo Alto, CA)^(5)^ and cone‐beam CT (CBCT), have recently become common. However, these methods often require additional cost, exposure of X‐rays, and longer time for setup. As a result, for a large number of patients who do not require a high accuracy of patient positioning (e.g., palliative treatments or mantle field radiation), portal imaging once or twice during the first week of treatment is still desirable.

The standard patient treatment regime employed at our institution involves first obtaining a CT scan of the patient which will be used for treatment planning. A DRR reconstruction of the beam's eye view (BEV) is computed and stored by a commercial treatment planning system (TPS). The treatment port, isocenter, orthogonal axes with scale information (a tick mark each centimeter) and other treatment information are burned into the DRR image.

Portal images are usually obtained at our hospital before the first treatment fraction using a megavoltage X‐ray source from a linear accelerator and a computed radiograph (CR) system (Fuji Medical Co., Ltd., Tokyo, Japan). Images are captured onto photosensitive plates, which are scanned to produce high resolution (usually 1760×1760 pixels) deep bit (10 bits per pixel) images. The portal images also contain scale information on the orthogonal axes with a small dot each centimeter. The portal and DRR images are compared to determine that the treatment beam accurately targets the planned treatment volume (PTV) while avoiding organs at risk (OARs). If a problem is detected, the presiding physician may request that the couch position be adjusted. Portal images are then retaken and rechecked against the DRR.

We developed an image registration software application for the estimation of patient setup error. DRRs from any commercial TPS can be opened using common file formats (e.g., bitmap, JPEG, DICOM). The software works for any anatomical region or gantry angle. The software can be operated manually, or with an automatic registration mode based on the mutual information between the images.

The purpose of this study is to verify that our software actually improves setup error detection compared with the traditional side‐by‐side method. There is little statistical evidence in the literature of the superiority of image registration to side‐by‐side human estimation of setup error from two‐dimensional portal images. We evaluated the efficacy of the software as an aid for the clinical staff to improve setup accuracy using a prospective phantom study and its statistical analysis. We compared the ability of humans to correctly determine a known setup error with and without the software. An automatic mode of operation of the software was also tested with a database of clinical cases, collected over several years, for which the setup up error was determined by the consensus of a trained software operator (medical physicist), a radiation technician and the presiding oncologist.

## II. MATERIALS AND METHODS

### A. Patient setup error detection software

After a CT scan of the patient is obtained, a DRR of the BEV is computed and stored with a TPS. The treatment port, isocenter, orthogonal axes with scale information (a tick mark each centimeter) and other treatment information are burned into the image. The DRR image is saved in a database accessible to our software. Portal images are usually obtained before the first treatment fraction using a double exposure (open field and the actual field) with a megavoltage X‐ray source from a linear accelerator, captured with a CR system. The portal images also contain graphical scale information on the orthogonal axes with a small dot or tick mark every centimeter from the isocenter.

In our proposed method, while the patient is lying on the treatment couch, the planning DRR and portal images are loaded into the software. The isocenter and two points on orthogonal axes, usually 10 cm from the isocenter, are designated by clicking on the images with the mouse. The locations of the points are determined by the operator using the axis and scale tick marks on each image. The software uses the three control points to determine scaling and rotation in order to transform the portal image into the coordinate space of the DRR image. Because the imaging plate may not be exactly orthogonal to the beam axis, especially when an oblique gantry angle is used, the portal image may be warped. The software can correct for these out‐of‐plane rotations.

**Figure 1:**
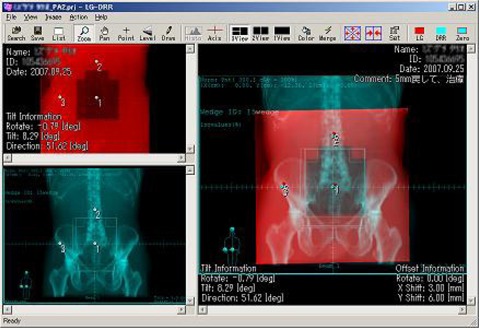
The Portal‐DRR software. The user specifies three points on the portal (top left) and DRR (bottom left) images using grid spacing tick marks. The merged image (right) is used to compare internal bony structures in order to determine the setup error, which is displayed at the lower right.

After the images have been successfully fused, the operator uses bony landmarks or other visible anatomical features in order to determine setup error. The portal image can be shifted horizontally or vertically and rotated clockwise or counterclockwise relative to the stationary DRR image. An example screenshot is shown in Fig. 1.

The operator can apply complementary colors, such as red and cyan, to make the fused image more distinguishable. If the images align exactly, each resulting pixel will be a level of grey. Misalignments are visible as color shadows. The operator can specify which pair of complementary colors to apply, depending on which colors are easier to see. Brightness and contrast can also be adjusted on both images to make bony features more distinguishable. The user can also rapidly flip between the DRR, portal and fused images. After the images are successfully registered, the treatment field shift (in mm) and rotation (in degrees), corresponding to the BEV, is displayed. The required couch movement to correct the offset, based on the gantry angle, can also be computed and displayed.

### B. Theory of image distortion correction

Distortions of portal images may include rotations about the beam axis (z‐axis) and rotations about an axis perpendicular to the beam axis (x‐ or y‐axis). It is difficult to independently correct rotations about the x and y axes. We therefore devised a scheme to treat rotations about the x and y axes as equivalent rotations about an arbitrary axis perpendicular to the z‐axis, denoted as the x′‐axis. The x′‐axis lies in the x, y plane and points in the direction of the tilt. It is possible to compose rotations about the x′‐axis from equivalent rotations about the x and y axes, combined with a rotation about the z‐axis. Coordinates on the imaging plate (x′, y′) correspond to the original coordinates (x, y) as follows:

(1) $$\left(\begin{array}{l} x^{\prime }\\ y^{\prime } \end{array}\right)=b\cdot Rot(\theta )\cdot Rot(\varphi )\cdot St(\phi )\cdot Rot^{-1}(\varphi )\left(\begin{array}{l} x\\ y \end{array}\right)$$

Here *b* is the zoom ratio, *Rot(*θ*)* is the rotation about the z‐axis, Rot(ϕ) is the rotation about the x'‐axis, and St(Θ) is the stretch ratio produced by the rotation about the x‐axis. The rotation and stretch functions are defined as:

(2) $$\text{Rot}(\theta )=\left(\begin{array}{ll} \cos\theta & -\sin\theta \\ \sin\theta & \cos\theta \end{array}\right),St(\phi )=\left(\begin{array}{ll} 1 & 0\\ 0 & \frac{1}{\cos\phi } \end{array}\right)$$

One can obtain the original image pixel coordinates by applying the reverse process in order to correct the distorted image:

(3) $$\left(\begin{array}{l} x\\ y \end{array}\right)=\frac{1}{b}\cdot Rot(\varphi )\cdot St^{-1}(\phi )\cdot Rot^{-1}(\varphi )\cdot Rot^{-1}(\theta )\left(\begin{array}{l} x^{\prime }\\ y^{\prime } \end{array}\right)$$

Because parameters θ, ϕ, and Θ can be obtained from two arbitrary coordinates, according to the equations, we can proceed as follows.

(4) $$\begin{array}{l} \phi =\text{arctan}\left(\frac{1}{2}\left(\alpha +\sqrt{4+\alpha^{2}}\right)\right)\;\;\;\;\varphi +\theta =\text{arctan}\;\;\left(\frac{m\cos\varphi +n\sin\varphi }{k\cos\varphi +l\sin\varphi }\right)\\ \varphi =\cos^{-1}\left(\frac{m\cos\varphi +n\sin\varphi }{k\sin\varphi -l\cos\varphi }\right)\;b=\frac{\cos(\varphi +\theta )}{k\cos\varphi +l\sin\varphi } \end{array}$$

Note that k, l, m and n are in the original beam axis' coordinate system and can be computed from two pairs of points.

(5) $$\left(\begin{array}{ll} k & l\\ m & n \end{array}\right)=\left(\begin{array}{ll} x_{1} & x_{2}\\ y_{1} & y_{2} \end{array}\right){\left(\begin{array}{ll} x_{1}^{\prime } & x_{2}^{\prime }\\ y_{1}^{\prime } & y_{2}^{\prime } \end{array}\right)}^{-1}=\frac{1}{x_{1}^{\prime }y_{2}^{\prime }-x_{2}^{\prime }y_{1}^{\prime }}\cdot \left(\begin{array}{ll} x_{1}x_{1}^{\prime }+x_{2}y_{1}^{\prime } & x_{1}x_{2}^{\prime }+x_{2}y_{2}^{\prime }\\ y_{1}x_{1}^{\prime }+y_{2}y_{1}^{\prime } & y_{1}x_{2}^{\prime }+y_{2}y_{2}^{\prime } \end{array}\right)$$

**Figure 2:**
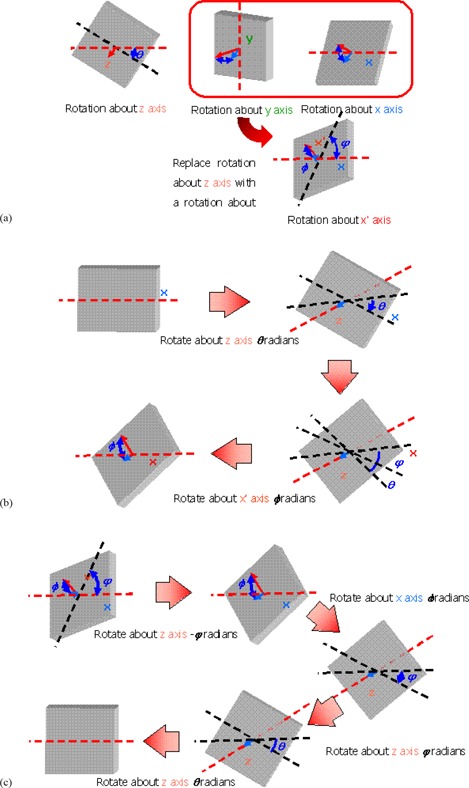
Conversion of rotation axis for image distortion.

The conversion process is illustrated in Fig. 2.

The proposed image‐registration method uses imaging plates to obtain portal images for irradiation field verification. For non‐zero gantry angles, the imaging plate is held freely on a stand without fixation to a certain coordinate system. In order to correct perspective distortion due to the imaging plate not being orthogonal to the beam axis, three control points are manually placed using graphical axis scale tick marks on each image. The specified points should cover the area of the image containing bony structures used for alignment. We refer to the area roughly surrounding the control points as the “working area”.

**Figure 3:**
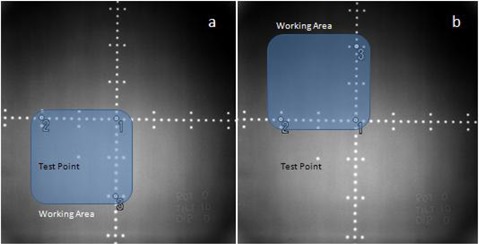
Tilt experiment setup. The imaging plate was tilted 10° horizontally. The test point (5 cm along the x‐ and y‐axis) was inside the working area for (a) and outside the working area for (b). The test point was transformed into the DRR image coordinates and the distance from the corresponding point specified on the DRR image was measured.

To test the correction function, an electronic tiltmeter was used to measure a 10° tilt of the imaging plate about the horizontal axis. The plate was exposed to X‐rays using the linac axis grid. The resulting image was fused with a dummy DRR image. A test point was placed at a position 5 cm along the x‐ and y‐axis within the working area. The distance from the test point on the DRR image and the transformed portal image point was measured. The experiment was repeated with the test point outside the working area. The experimental setup is illustrated in Fig. 3.

### C. Automatic determination of patient setup error

We developed a method to automatically determine the x‐y shift and in‐plane rotation based on the mutual information (MI) of the pixel intensity values of the overlapping portions of the transformed portal and DRR images. The entropy of a single image can be computed from the equation:

(6) $$H=\sum_{i}p_{i}\cdot \log\frac{1}{p_{i}}$$

where $p_{i}$ is the probability of a gray‐level pixel value *i* estimated from the histogram of the image.^(6)^ The joint entropy between two images can likewise be defined as:

(7) $$H(A,B)=-\sum_{i,j}p(i,j)\cdot \log[p(i,j)]$$

where *p(ij)* is the probability of gray‐level pixel value *i* from one image (A) and the gray‐level pixel value *j* from the second image (B) at the same position. Mutual information is then defined as:

(8) I(A,B)=H(A)+H(B)−H(A,B)

where *H(A)* is the entropy of the first image (i.e., the portal image), *H(B)* is the entropy of the second image (i.e., the DRR image), and *H(A,B)* is the entropy of their joined histogram.^(7)^

Optimizing the MI involves finding the x‐y shift position that yields the highest mutual information coefficient, automatically shifting the portal image left, right, up and down a determined step size, and choosing the position with the highest MI coefficient. If the current x‐y shift value yields the highest MI coefficient, the optimization loop is abandoned. In order to reduce the likelihood of stopping at a local maximum, we employ a multiresolution method. The initial step size is relatively large, usually 4 mm. The best MI coefficient is located with the initial step size. The step size is then reduced by half and the process is repeated. The final step size is usually 1 mm, but can be set lower for submillimeter accuracy.

Adding rotation checks can increase optimization time exponentially if every possible rotation is checked at each shift location. However, if we assume that the setup rotation is small, a good compromise approach is to search rotation and shift iteratively; that is, search for the best x‐y shift and then search for the best rotation. This process is repeated until the best possible rotation is found at the best possible x‐y shift.

### D. Phantom study

CT scans of anthropomorphic thorax and pelvis phantoms were taken. The images were read into a commercial treatment planning software system (Xio; CMS, Inc., St. Louis, MO). DRR images (one for the thorax, one for the pelvis) were generated using an antero‐posterior (AP) port with a 10×10 cm square field.

**Figure 4:**
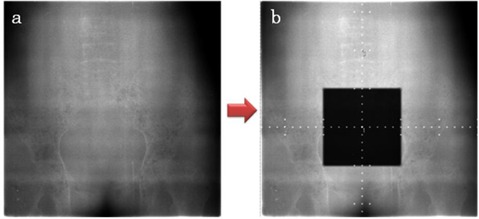
Portal image taken without field or scale tick marks (a). Automatically generated treatment field and scale ticks applied to the phantom portal image (b).

In order to be able to present a portal image with a known but arbitrary x‐y error offset, we first obtained a single portal image of the phantom without field or scale ticks. Our test software burns in the treatment field and tick marks based on determined horizontal and vertical offset error values (Fig. 4). The portal images of the phantoms were acquired with a 6 MV photon beam from a linear accelerator. A list of five cases was prepared with offset values between plus and minus 10 mm horizontally and vertically. Rotation was not considered in this study. In order to determine each subject's consistency in determining the offset error, each case was presented twice for a total of ten cases. Each subject was presented with the same offset cases, but the order of the case list was shuffled.

For the ten thorax and ten pelvis cases generated by the computer with the above method, we presented the series of cases to each examiner using three methods. First, five licensed radiation oncologists determined setup error with the conventional side‐by‐side method, without the aid of the registration software. We refer to this as the “side‐by‐side method”. These doctors included experienced physicians, as well as doctors in training. Four radiation technicians were then asked to determine the same series of setup errors using the registration software, which we refer to as the “registration method”. The automatic determination function of the software was also used to determine the setup error of the same case list (“automatic method”). Doctors and technicians were allowed to adjust image brightness and contrast. No time constraint was in place during the testing.

### E. Statistical analysis

The distance from actual and estimated offset was computed with $\sqrt{{\left(x-x_{0}\right)}^{2}+{\left(y-y_{0}\right)}^{2}}$ where (x, y) is the estimated offset and $\left(x_{0},y_{0}\right)$ is the actual offset. The average and standard deviation of the distance was computed for each examiner.

The average consistency of an examiner was defined as the average geometric distance between two estimations of a single case. The mean of the methods was compared with the paired t‐test. The difference of the average consistency among the three methods was also computed in order to determine if consistency improved using the software. All statistical analysis was performed with JMP version 8 (SAS Institute Inc., Cary, NC).

### F. Database study

In order to test the performance of the automatic registration mode of the software on actual clinical data, we assembled a database of over 5,000 patient setup cases performed between April 2007 and December 2009 at our hospital. At the time of treatment, the portal image for each case was registered with the corresponding DRR image using the manual mode of the software, and the setup error for each case was determined by a consensus of three people: a software operator (medical physicist), a radiation technician, and the attending oncologist. Although it is difficult to establish a “gold standard” for clinical data, for this study we assume the human‐determined offset is correct, or at least very near the actual offset error.

After assembling the database, a large batch script was executed to open each case, fuse the portal and DRR images, and automatically determine the error offset using the MI optimization algorithm described above. The automatically‐determined error offset was then compared with the human‐determined error offset. The geometric distance between the two values was computed and a statistical analysis was performed.

## III. RESULTS

### A. Phantom study

The results of the thoracic and pelvis are shown in Fig. 5(a) and (5b), respectively. The geometric distance from the actual offset and the determined offset is plotted. Results from five doctors (DR1–DR5) using the side‐by‐side method, four technicians (TECH1–TECH4) using the registration software in manual mode, and the automatic method (COMP) are shown.

**Figure 5(a):**
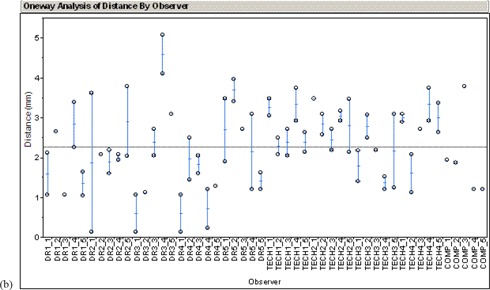
Results of AP chest study.

**Figure 5(b):**
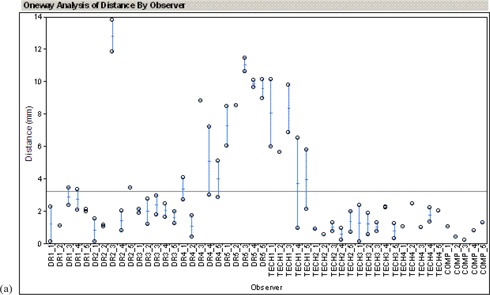
Results of AP pelvis study.

For the thorax, the average distance between the actual setup error and the estimated error was 4.3±3.0 mm for the radiation oncologists without the registration software, 2.1±2.4 mm for technicians with the registration software, and 0.8±0.4 mm for the automatic algorithm. For the pelvis, the average distance between the actual setup error and estimated error was 2.0±0.5 mm for the radiation oncologists without the registration software, 2.5±0.4 mm for technicians with the registration software, and 2.0±1.0 mm for the automatic algorithm.

Each case was presented to the examiner twice. These two values are plotted vertically for each examiner. Consistency was measured as the geometric distance between the two estimated offsets of the same portal image. This corresponds to the height of the vertical bar in Fig. 5. The results are summarized in Table 1.

**Table 1:** Average distance from estimated and actual setup error.

*Phantom*	*Method*	*Average Distance*
	Side‐by‐side	4.3±3.0 mm
Chest	Registration	2.1±2.4 mm
	Auto	0.8±0.4 mm
	Side‐by‐side	2.0±0.5 mm
Pelvis	Registration	2.5±0.4 mm
	Auto	2.0±1.0 mm

In order to determine statistically whether setup error estimation improved using the software, we computed the average consistency among the three methods. The average consistency for the side‐by‐side method was 2.4±2.0 mm for thorax and 1.4±1.2 mm for pelvis. The average consistency for the registration method was 1.7±1.6 mm for thorax and 0.9±0.5 mm for pelvis. The results are summarized in Table 2. Note that the computer algorithm always produces the same result for a given input, so it is perfectly consistent.

**Table 2:** Average consistency of test subjects.

*Phantom*	*Method*	*Average Consistency*
Chest	Side‐by‐side	2.4±2.0 mm
	Registration	1.7±1.6 mm
Pelvis	Side‐by‐side	1.4±1.2 mm
	Registration	0.9±0.5 mm

In order to test statistically that setup error estimation improved using the software, we computed the paired two samples for means. The null hypothesis is that population mean of the differences between the paired values is zero. The results are summarized in Tables 3(a) and 3(b).

**Table 3(a):** Comparison of estimation methods (chest).

*Comparison*	*p‐value*
Side‐by‐side ‐ Registration	0.0067
Side‐by‐side ‐ Auto	0.0002
Registration ‐ Auto	0.0001

**Table 3(b):** Comparison of estimation methods (pelvis).

*Comparison*	*p‐value*
Side‐by‐side ‐ Registration	0.0047
Side‐by‐side ‐ Auto	0.4547
Registration ‐ Auto	0.0593

### B. Database study

Results of the database study are summarized in Table 4. Overall, the automatic registration method does not perform well at around 7 mm from the human‐estimated offset. However, for certain anatomical regions, such as head and breast, the algorithm consistently estimated the setup error within about 2 mm of the human estimation. Automatic registration was especially poor for pelvic regions — more than 1 cm on average. This is probably due to the lack of contrast in the portal images.

**Table 4:** Comparison of auto‐shift function with clinical case database.

*Group*	*Count*	*Mean Dist. (mm)*
All	5101	7.0±2.1
AP Head	407	2.4±2.1
LR Head	537	2.2±2.7
AP Neck	158	5.5±6.1
LR Neck	214	4.2±4.3
OB Neck	95	4.8±4.1
AP Chest	264	5.1±4.5
LR Chest	101	7.7±8.0
OB Breast	160	3.6±2.7
AP Pelvis	148	13.8±19.8
LR Pelvis	148	12.8±11.7

In order to demonstrate the usefulness of the automatic mode of operation for two sample anatomical cases, we arbitrarily divided the distance between the human and computer estimated offsets into four bins: ≤ 2 mm (Good), ≤ 5 mm (Fair), ≤ 10 mm (Poor) and > 10 mm (Terrible). Tables 5(a) and 5(b) show the results for head and pelvis cases for all gantry angles.

**Table 5(a):** Comparison of auto‐shift function with humans (head).

*Bin*	*Frequency* (total=61)
Good (≤2 mm)	35 (57%)
Fair (≤5 mm)	23 (38%)
Poor (≤10 mm)	1 (2%)
Terrible (>10 mm)	2 (3%)

**Table 5(b):** Comparison of auto‐shift function with humans (pelvis).

*Bin*	*Frequency* (total=48)
Good (≤2 mm)	4 (8%)
Fair (≤5 mm)	9 (19%)
Poor (≤10 mm)	16 (33%)
Terrible (>10 mm)	19 (40%)

**Table 6:** Result of tilt experiment.

*Point Placement*	*Distance*
Inside working area	0.7 mm
Outside working area	1.5 mm

### C. Tilt experiment

Results of the tilt experiment are presented in Table 6. When the test point was placed 5 cm within the working area, the distance from the DRR point and the transformed portal image point was 0.7 mm. When the point was placed 5 cm outside the working area, the distance increased to 1.5 mm. This result indicates that the user must be careful to consider only bony structures within the working area when the out‐of‐plane tilt is large. The automatic registration algorithm should also ignore pixels outside the working area.

## IV. DISCUSSION

Our results show that the registration method was at least not worse than the side‐by‐side method, and the automatic method was statistically better than both the side‐by‐side method and registration method for the thorax phantom case studied. This result suggests that our software can be a reasonable complementary method in the clinical practice. Based on this study, the software has been installed in our hospital information system and has been used in clinical practice since April 2007. Moreover, the time required for the estimation of the setup error has not been extended by the usage of this program, once the operator obtained sufficient experience with the software.

A number of criteria influenced our software design decisions:

- Because image registration is performed while the patient is waiting in the treatment position, the system must be fast — less than about one minute after the portal image is obtained.
- DRRs by any commercial planning software are to be used, as long as scale information is burned into the bitmap. The DRR image can even be a screen‐captured bitmap, or a digital scan of a paper, or film print.
- Any common file format (e.g., bitmap, JPEG, DICOM) can be used for either the portal or DRR image.
- The system must work with any beam view of any anatomical region encountered in clinical practice.
- The software must run on a single standard PC running the Windows operating system.

We have found that there was a large inconsistency in the side‐by‐side method among doctors. This is probably due to a difference in training or experience. The large variation in the accuracy of the final decision based on the portal film may influence the clinical outcome. The improvement in the consistency with automatic registration (which was found in this study for thorax) may improve the local control rate and complication rate in this context.

If Dr. 5, in particular, is omitted, the side‐by‐side method compares well with the registration methods. Although Dr. 5 was a licensed radiation oncologist with experience estimating setup errors, he or she may have needed more practice with our experimental setup. Although we explained that the movement of the radiation field (rather than the treatment couch) should be specified, it is easy to mistake left‐right or up‐down shift. We feel that these kinds of human errors are inevitable when relying solely on human judgment with the side‐by‐side method.

From the database study, we can state that the automatic mode of operation performs well for head, neck and breast cases, but performs poorly for pelvic cases. Due to the thickness of the human anatomy in the pelvic and abdomen regions, the contrast of the resulting megavoltage portal image is very low. This makes automatic registration based on mutual information extremely difficult. In order to improve the performance of the automatic registration, the contrast of the portal image needs to be improved. Possible methods of improving portal image contrast include using kilovoltage X‐ray, or using advanced digital image processing.^(8)^ Other methods, such as restricting the area used to compute the mutual information may further improve auto‐registration.

Although the automatic registration algorithm performed reasonably well on the pelvis phantom, the brightness and contrast (window level) of the portal image from the phantom image was adjusted by hand to maximize the contrast of the pelvic bone. In the database study, however, it was not feasible to adjust all cases by hand. The window level was computed automatically from the raw scanned data based on the histogram of pixel values. If the window level algorithm can be improved, the image contrast may be increased. Sophisticated image filters may also improve the signal‐to‐noise ratio and make bony features more recognizable.^(9)^ We hope that this will, in turn, improve the auto‐registration results.

While some verification systems are applicable to only a particular application (for example, lung cancer^(10)^ or pelvic treatments^(11)^), our system is used for all cases encountered in our practice, including oblique views. Some systems require the computation of custom DRR images in order to match as closely as possible the portal image.^(12)^ Our system uses the DRRs that are generated by a commercial TPS software package. Thus, the same pair of portal and DRR images that the attending physicians have normally been using are used by our software.

Some systems require the user to draw bony structures on the source images.^(13)^ While this is an option with our software, it is not required. It is sometimes useful to draw the outline of a bone on both images, and then see how closely they align on the merged image; however, we found that this method significantly increases the burden on the operator. It is generally faster to view the merged portal‐DRR image without hand‐drawn contours.

Although EPID has become popular, some EPID systems are quite expensive to maintain and have reduced imaging quality. Our software can be used in many situations where good quality EPID is not available.

Because our system uses only three points to calculate the out‐of‐plane transformation, areas far from the control points (outside the working area) may not align exactly. If the entire image is to be used for alignment, an alternative method would be to use a calibration function such as McalList from the Matrox Imaging Library.^(14)^ With this method, a list of corresponding points on the portal and DRR images is passed to the function and a perspective distortion correction matrix is calculated. The more points that are specified, the more accurate the mapping. However, because only points on the axes can be specified, the corners of the corrected portal image may still be slightly distorted. Specifying many points may also be a burden on the user. An automatic method of detecting all the visible grid tick marks on the portal and DRR images is under investigation.

## V. CONCLUSIONS

The ability to estimate offset values improved using our software for the chest phantom that we tested. Setup error estimation was further improved using our automatic error estimation algorithm. Estimations were not statistically different for the pelvis case. Comparing the automatic setup function with a database of clinical cases estimated by human operators revealed that the automatic function works relatively well for head, chest and breast cases, but performs poorly for pelvis and other cases. Automatic registration should improve by increasing the contrast of the portal image. Although setup error can be manually judged accurately and quickly with the software as an aid to doctors and technicians, work remains to make the software more fully automatic.
